# Two decades of tobacco use prevention and control policies in Cameroon: results from the analysis of non-communicable disease prevention policies in Africa

**DOI:** 10.1186/s12889-018-5828-4

**Published:** 2018-08-15

**Authors:** Clarisse Mapa-Tassou, Cecile Rénée Bonono, Felix Assah, Jennifer Wisdom, Pamela A. Juma, Jean-Claude Katte, Zakariaou Njoumemi, Pierre Ongolo-Zogo, Leopold K. Fezeu, Eugene Sobngwi, Jean Claude Mbanya

**Affiliations:** 1 Department of Public Health, Faculty of Medicine and Biomedical Sciences, University of Yaoundé I, Yaoundé, Cameroon; 2 Health of Population in Transition Research Group (HoPiT), Yaoundé, Cameroon; 3 University of Yaoundé II, Yaoundé, Cameroon; 4 Center for Development of Best Practices in Health (CDBPH), Yaoundé, Cameroon; 5 Wisdom Consulting, New York, NY USA; 6 African Population and Health Research Center (APHRC), Nairobi, Kenya; 7 Université Paris 13, Equipe de Recherche en Epidémiologie Nutritionnelle (EREN), Centre de Recherche en Epidémiologie et Statistiques Sorbonne Paris Cité, Inserm (U1153), Inra (U1125), Cnam, COMUE Sorbonne Paris Cité, F-93017 Bobigny, France

**Keywords:** Health policy analysis, Tobacco control and prevention, Cameroon

## Abstract

**Background:**

Tobacco use is the leading cause of preventable death in the world today. In 2010, the World Health Organization (WHO) proposed efficient and inexpensive “best buy” interventions for prevention of tobacco use including: tax increases, smoke-free indoor workplaces and public places, bans on tobacco advertising, promotion and sponsorship, and health information and warnings. This paper analyzes the extent to which tobacco use prevention policies in Cameroon align with the WHO tobacco “best buy” interventions. It further explores the context, content, formulation and implementation level of these policies.

**Methods:**

This was a case study combining a structured review of 19 government policy documents related to tobacco use and prevention, in-depth interviews with 38 key stakeholders and field observations. The Walt and Gilson’s policy analysis triangle was used to describe and interpret the context, content, processes and actors during the formulation and implementation of tobacco prevention and control policies. Direct observations ascertained the level of implementation of some selected policies.

**Results:**

Twelve out of 19 policies for tobacco use and prevention address the WHO “best buy” interventions. Cameroon policy formulation was driven locally by the social context of non-communicable diseases, and globally by the adoption of the WHO Framework Convention on Tobacco Control. These policies incorporated at a certain level all four domains of tobacco use “best buy” interventions. Formulating policy on smoke-free areas was single-sector oriented, while determining tobacco taxes and health warnings was more complex utilizing multisectoral approaches. The main actors involved were ministerial departments of Health, Education, Finances, Communication and Social Affairs. The level of implementation varied widely from one policy to another and from one region to another. Political will, personal motivation and the existence of formal exchange platforms facilitated policy formulation and implementation, while poor resource allocation and lack of synergy constituted barriers.

**Conclusions:**

Despite actions made by the Government, there is no real political will to control tobacco use in Cameroon. Significant shortcomings still exist in developing and/or implementing comprehensive tobacco use and prevention policies. These findings highlight major gaps as well as opportunities that can be harnessed to improve tobacco control in Cameroon.

## Background

Tobacco use is a significant cause of morbidity and mortality [1]. Globally, 12% of all deaths among adults aged 30 years and over is attributed to tobacco use [2], and it is estimated that 80% of these deaths will be in low- and middle-income countries (LMIC) by 2030 [2]. Tobacco use is increasing rapidly in LMIC such as Cameroon, due not only to steady population growth with increasing urbanization and westernization of lifestyles, but also targeting by the tobacco industry ensuring that millions of people become addicted each year [3]. The country is undergoing a rapid socio-economic transition characterized by improving standards of living, rapid unplanned urbanization, and westernization of lifestyles, including increased tobacco use, harmful use of alcohol, unhealthy diet and insufficient physical activity levels. The result is an epidemiological transition in which non-communicable diseases (NCDs) increasingly contribute a significant share of the public health burden of disease [4]. In Cameroon, tobacco use is common in all forms including chewing, sniffing, and especially cigarette smoking. Increasing numbers of women are using tobacco [5, 6]. In 2003, in the Cameroon Burden of Diabetes Baseline (CAMBoD) survey, overall 6.3% of the total population declared to be current smoker [7]. In addition to active smoking, passive smoking is recurrent; nearly one-third of the population said they were affected by second-hand smoke: in 1994, 35.7% of Cameroonians were exposed to it, while the report of the Ministry of Public Health noted a slightly higher percentage (37%) in a study carried out in 2006 [8]. The 2013 Global Adult Tobacco Survey on people above 15 years of age showed that 13.9% of men and 4.3% of women used tobacco products; 5.7% of adults currently smoked cigarettes [5]. On the Global Youth Tobacco Survey done in 2008 and 2014 among school children aged 13 to 15 years, 5.7% currently smoked cigarettes [6, 9].

To address the burden of tobacco use, World Health Organization (WHO) recommends the adoption and implementation of the Framework Convention on Tobacco Control (FCTC) and at least, WHO tobacco prevention “best buy” interventions. These “best buy” interventions include: tax increases; smoke-free public spaces; ban of tobacco advertising, promotion and sponsorship; and health information and warnings. Recently, Cameroon has begun to recognize NCDs as a public health threat [4, 10, 11] and some actions have been undertaken for the control of NCDs and their risk factors, including addressing tobacco use. A clear understanding of the context and processes of formulation of policies and their implementation is necessary to ensure effective interventions to prevent and control tobacco use. Although there are efforts worldwide to evaluate the impact of tobacco policy on tobacco use [12], this is the first study of tobacco control policies in Cameroon.

This paper analyzes the history of tobacco use prevention and control policies in Cameroon. First, it explores the extent to which tobacco use and prevention policies in Cameroon align with the WHO tobacco “best buy” interventions. Secondly, it describes the context, the content, the formulation and implementation process of these policies and their effectiveness.

## Methods

### Study design

We used a qualitative case study design [13] with Cameroon’s adoption of tobacco use prevention “best buy” interventions constituting the case [14]. This qualitative design helped us to explore in-depth the context, content, processes and actors of formulation and implementation of tobacco control policies as well as to assess the facilitators and barriers faced by actors in formulating and implementing policies [15].

### Guiding framework

We used the Walt and Gilson’s framework to analyze Cameroon tobacco control policies. This framework acknowledges the non-linearity of the policy process as well as the incremental nature of policy making and focuses on the analysis of policy context, content, actors and processes [15, 16]. The context may include changes in the political climate and management structures; socio-cultural, economic or technological changes; changes in the global financial situation; and conflicting development programs between governments and development partners. The content of a policy can be analyzed by examining the policy objectives, the way it’s designed, whether it has an implementation plan and specific mechanisms for updating, if needed. Political actors were assessed by verifying those who were involved in the political processes, their roles and position(supportive, mixed or non-supportive) and who else should have been involved. Processes include the different stages of the policy process and the strategies used to involve different actors.

### Data collection procedures

Data was collected through three techniques: document review, in-depth interviews and direct observations.

#### Document review

The aim of the document review was to describe the Cameroon policy context and content, identify existing policies and gaps therein and understand the policy development processes and implementation status. We focused on policy documents on tobacco use prevention and control (including acts and laws, strategic plans, guidelines and government directives), relevant documents on tobacco use and reviews and case studies of successful policy formulation and implementation at national level. All documents review was done between mid-2014 to the end of 2015 in which we selected related sectors based on their expected role in tobacco use policy formulation and implementation. We looked for laws, presidential decisions, ministerial orders, circulars, recommendations and guidelines, action plans, articles, government structures, research institutions, NGOs and associations of civil society reports from both online databases and in physical space. An initial screening of all the selected tobacco policy documents was performed by the primary investigator (CMT). This was followed by a second review by a secondary investigator (CRB) after which documents not relating to the WHO “best buy” interventions were eliminated.

We extracted data from retrieved documents and entered them into a Microsoft Excel database by these variables: authors, document title, date of publication, publisher/journal title, source, tobacco policy element addressed, summary of document type and objective, summary of key document analysis findings related to “best buy” interventions.

#### In-depth interviews

We conducted interviews designed to understand the perspectives of key policy-makers and implementers on formulating and implementing current tobacco policies, as well as the strengths and weaknesses of Cameroon’stobacco policies development. We used a combination of purposive and snowball sampling [17] to select key informants about prevention policies and their formulation and implementation. We first created a list of potential key contact(s) from the department or office in the sector responsible for the identified policy using information available in the public domain as well as contacts of known key stakeholders. We then progressively identified more actors and sectors using a snowball technique by asking the key contact person to identify other actors and sectors involved in the policy. We repeated these steps for all identified policies.

We used different interview guides for policy-makers and policy implementers. The policy makers’ interview guide focused primarily on the context, process and actors of formulation. Whereas for the implementer’s interview guide, we focused more on the process and actors of the implementation.We limited our interviews to actors of national policies formulated only in the last 10 years both because of our focus on formulation and to reduce possible recall bias and availability of actors for policies implemented more than 10 years ago.

During interviews, we collected data on: (1) the policy context, including the political and historical context, social and economic factors, the health system, and technological factors; (2) the policy content, including risk factors addressed in the policy, the rationale for the policy, the objectives of the policy, the actors in the policy, other interventions mentioned, and mechanisms of actualizing the policy; (3) policy formulation and implementation processes; and (4) actors in the policy formulation and implementation as well as facilitating and hindering factors in their involvement. All interviews were recorded on a digital recorder and lasted 60 to 90 min. After each interview, the research team listened to the recorded conversations and ensured that note-taking was accurate. Once interviews were transcribed, another member listened again to check its quality. We asked a sample of key informants to validate their transcripts.

#### Observations of the effectiveness of policy implementation

In order to identify the extent to which policies were implemented, we conducted systematic observations in 5 of the 10 regions of the country. We selected four observable tobacco use prevention “best buy” interventions: ban of tobacco advertising, marking of health warnings on packages of tobacco products, creation of smoke free zones and prohibition of sales, and distribution and consumption of tobacco products in secondary schools. For each policy and in each region, we developed an observation guide, selected a number of sites situated at the main roads and avenues and observed without any interaction with community members. For example, the observation guide looked for the existence of tobacco advertising on official public billboards or other places and the existence of a posted sign with the inscription“college X/high school X is a smoke-free zone” at the main entrance of educational institutions. These observations helped us to triangulate with data collected during interviews with key informants and during document review about the extent of implementation.

For interviews and observations described above, we selected 5 of the 10 regional capital cities to span the 5 different socio-ecological zones of the country: Yaoundé, Douala, Ebolowa, Bamenda and Garoua.

### Data management

Using the Walt and Gilson framework [16], we developed a comprehensive codebook to guide coding of documents, interviews, and community observation reports. Codes identified the content, context, and process of tobacco prevention and control “best buy” interventions categories as well as the individual, group, and organization actors involved.

We conducted data collection and analyses concurrently. We prepare a data analyses plan and compiled all datasets including the spreadsheets of policies, transcripts and field notes from interviews, and notes from observations. We cleaned data by reading through the transcripts to identify incomplete sections, typographical errors, formatting errors and clarifying the use of idioms, metaphors and slang language. Notes were inserted where explanations were required before the data was uploaded into NVivo10 software [18] for coding and analysis. Most interviews (*N* = 33; 87%) were conducted in French. Transcriptions, coding, and preliminary analyses were done in French and then translated into English for this manuscript.

### Data analyses

We analyzed qualitative data using a content analysis approach. Researchers conducted content analysis using the NVivo10 software applying a pre-determined coding frame based on Walt and Gilson’s framework. The software assisted researchers in labeling sections of text with content areas and key themes, from which we performed a thematic analyses [19]. We sorted codes into categories depending on how the different codes were thematically related and linked. We also integrated data across data sources by examining areas of consistency and inconsistency among findings.

### Ethical considerations

Interview participants were informed about the nature of the study, the potential risks and benefits of the study, confidentiality, and their right to withdraw from the study at any time without penalty. The interviews were conducted at times and venues mutually agreed upon by the research team and key informants. These venues were free from distractions and security risks. We ensured all venues were private places where the conversation could not be easily overheard by others. Participants provided written consent to take part in the study. The National Ethics Committee of the Cameroon’s Ministry of Public Health approved this study and provided ethical oversight.

## Results

### Overview of data sources

We identified 35 documents related to tobacco prevention and control in Cameroon, with 19 being tobacco prevention policies (Table 1). Twelve of the 19 policies included at least one of the WHO “best buy” interventions for tobacco control. We conducted interviews with 38 policy makers and staff responsible for formulating and implementing selected policies related to tobacco use “best buy” interventions (Table 2).

**Table 1:** Documents reviewed

Document type	Total number
National NCD policy documents related to “best buy” interventions	19
Published journal articles (Relating to tobacco in the country)	6
Other articles related to tobacco	3
Reports from international organizations	7
Total	35

**Table 2:** Type and number of informants by sector

Informants type by government sector	Number	% (sector)
Education	23	60.5
Communication/information	5	13.1
Trade and Industry	4	10.5
Health	2	5.3
Finance	4	10.6
Total	38	100

### Policy context

The introduction of the tobacco control discussions to the political agenda of Cameroon was influenced by both global context and local factors. High tobacco consumption and its effects were global concerns that led to international coalition towards tobacco control. In 2003, the FCTC was developed and ratified by Cameroon in 2006. The adoption of this convention motivated the formulation of various policies in Cameroon such as health warnings on tobacco products and creation of smoke-free zones.

With regard to the local context; the formulation of tobacco control policies was driven by epidemiological data on tobacco use. Most decisions in Cameroon are based on its adverse effects on humans with an emphasis on the problem of smoking/delinquency among youths and particularly in school settings. In Cameroon, studies showed that current smoking status is associated with male sex, parental smoking, family smoking and friends smoking [20–22]. The socio-professional category of people working in the informal sector had a prevalence of active smoking higher compared to other categories. Living with smokers is a fact classically described as a risk factor of tobacco consumption. People living in the same household with smokers in Cameroon are more than 12 times more likely to be smokers than those who lived in a home without smokers [22].

Another factor was the health system reform. In 2001, the government of Cameroon started to integrate non-communicable diseases in its health policy agenda. This led to a re-organization of the Ministry of Public Health by the Head of State and the creation of two new departments; the Department of Health Promotion and the Department of Disease Control, the latter having a sub-department for non-communicable diseases [23]. In the department of health promotion, a national committee was created with main purpose to deal with drugs control and tobacco use prevention and control.

Economic factors also influence formulation of tobacco policies. Cameroon is a tobacco-producing country [8] and government policy took into account that increased tobacco production benefits economic development. The tobacco industry consists mainly of the production of raw tobacco leaves, the industrial production of cigarettes and the importation of tobacco products [5, 8]. The Food and Agricultural Organization estimates that unmanufactured tobacco leaf production was steady at around 6000 tons per year between 2012 and 2014 [24]. In 2006, the Cameroonian government, aware that tobacco farming generated significant revenue for certain producers, approved a project called “PARTEC” [project supporting the revival of tobacco farming in eastern Cameroon]. Since 2010, this sector therefore has regular subsidies from the Public Investment Budget of the Ministry of Agriculture, in order to boost the national tobacco production [25]. This paradox of the State financially supporting tobacco farmers and at same time preventing and controlling tobacco use has been challenged by the civil society. During a Parliamentary session in 2014, the Prime Minister while answering a question said this paradox would be addressed within the context of the implementation of the WHO FCTC [26].

### Policy content and “best buy” interventions

The policy context, described above, led to the formulation of 12 tobacco control policies which incorporated the tobacco use prevention “best buy” interventions: tax increases on tobacco products, smoke-free indoor workplaces and public places, bans on tobacco advertising, promotion and sponsorship, and health information and warnings (Table 3).

**Table 3:** Timeline of development of tobacco policies addressing the WHO tobacco use prevention“best buy”interventions

Policy	Year of development	Lead sector	“Best buy” interventions addressed in the policy			
			Tax increase	Protect people from tobacco smoke	Warn about the dangers of tobacco	Bans on tobacco advertising
Decision N° 0222/P/MSP/SGF/DMSP prohibiting smoking in all structures of the Ministry of Health	1988	Health		x		
Law N° 98/004 on the orientation of education in Cameroon	1998	Education		x		
Law No. 2006/018 of 29 December 2006 governing advertising in Cameroon		Communication				x
Memo N° 1913 prohibiting smoking in the structures of the Yaoundé City Council	2007	Territorial administration		x		
Circular N° 07/788/CF/L/Finance Ministry / HR / SP prohibiting smoking in all structures of the Ministry of Economy and Finances	2007	Finance		x		
Order N° 967 Ministry of Public Health and Ministry of Trade, regarding health warnings on packages of tobacco products	2007	Health/Trade			x	
Circular N° 012/B1/1464/ MINEDUB /SG/HR/ SSSAPPS establishing non-smoking areas and anti-tobacco clubs in schools	2007	Education		x		
Circular N° 19/07/MINESEC / SG/HR/SDSSAPPS of on the establishment of anti-tobacco clubs in schools and making schools “non-smoking areas.”	2007	Education		x		
Circular N° MINESUP / SG / DPDSU on tobacco control in the central services of the Ministry of Higher Education and public universities	2012	Education		x		
Circular N°003 /LC/MINAS/SG/DSN/SDLCES/ SLCFS prohibiting smoking in all structures of the Ministry of social affairs	2014	Social Affairs		x		
Law N° 2014/026 of 23 December 2014 on the finance law of the Republic of Cameroon for the 2015 financial year	2014	Finance	x			
Sub-Prefectoral decision N° 06/SPD/BLPA/2015 prohibiting smoking in public places in Bamenda I Sub-Division	2015	Territorial administration		x		

The increase of tobacco taxes was identified as a powerful policy tool and historically the most cost-effective intervention to reduce smoking [27, 28]. In Cameroon, y the increase of excise tax was one of the interventions. Excise tax refers to an indirect type of taxation imposed on the manufacture, sale or use of certain types of goods and products. The *Ad valorem* excise tax, which is one type of the excise duty meaning that a fixed percentage is charged on a particular good or product, was introduced for tobacco products in 1999 as part of the Central African Economic and Monetary Community (CEMAC) regulation [29]. CEMAC countries then implemented the *Ad valorem* system excise duties applied at 25% on tobacco products., in Cameroon, the excise duty was applied at 25% with a minimum of not less than 2600 CFA francs (USD 4.37) for 1000 sticks of cigarette. In 2015, this policy was revised in the 2015 National Finance Act: the minimum of excise tax was increased from 2600 to 3500 CFA francs (USD 4.37 to 5.89) for 1000 sticks of cigarettes [30]. This resulted in an increase of 100 CFA francs per pack of 20 sticks of cigarettes in 2015.

Considering the “best buy” intervention on the creation of smoke-free indoor workplaces, we also found individual government ministries and departments policies integrated it [31–35] (Table 3). These included the Ministries of Basic Education, Secondary Education, Higher Education, Finance, and Social affairs, and also one territorial sub-division in the country’s North-West region [36]. For example, the circular letter formulated by the Ministry of Secondary Education instructs that a sign marked “college X/high school X or teacher’s school X is a smoke-free zone” must be posted at the main entrance of each facility, classrooms, office and commercial areas, orders that tobacco prevention activities with school staff and students must be organized, and requires the creation of anti-smoking clubs.

Several Cameroon policies incorporate the“best buy” intervention with health warnings printed on tobacco packages. Order N° 006/MINDIC/MSP/CAB/DU of 08 June 1999 ordered that tobacco products must carry the text “WARNING FROM THE MINISTRY OF PUBLIC HEALTH: TOBACCO MAY BE DANGEROUS TO YOUR HEALTH”. In 2007, this policy was updated and a conjoint ministerial order N° 967 of Ministry of Health and Ministry of Trade [37] was signed to warn tobacco users about the serious health effects of secondhand smoke. The updated policy stipulates that at least 50% of principal display areas on the front and the back of a retail package of any tobacco product should be covered by health warnings. This policy mandates the following specific health warning on cigarettes packages: “Tobacco causes serious health damages to the smoker and that of his surroundings”. This warning specifies font color (white), font size and type (16, times News Roman) and background color (black), must be written in French on one side of the package and English on the other side.

The fourth tobacco “best buy” intervention requires policies for a comprehensive ban on all forms of tobacco advertising, promotion and sponsorship. Cameroon passed such a law in the 2006 [38]. The policy stipulated that implementation should immediately follow promulgation and provided penalties for noncompliance; its article 62 stipulates a fine of 20,000,000 – 50,000,000 CFA francs (USD 33,690 – 84,175) on any person who, contrary to the provisions of articles 39, Art. 40 (1) and (2) of this law, shall cause a commercial message to be disseminated on cigarettes or other tobacco products.

### Policy process

The formulation of tobacco prevention policies started around 1988 and were propounded mainly by the health sector. In the 2000s, the international campaign against tobacco championed by WHO and the establishment of the FCTC in 2003, motivated local policy makers to review tobacco prevention policies, resulting in the intensification of these policies and interventions. Thus, in 2004, the Ministry of Public Health through the Department of Health Promotion, created a 15 person multisectoral expert group on tobacco called “GROUPE” (Decision No. 00615/D/MSP/DPS of 11 February 2004) [39]. The mission of the expert group was to brainstorm and conduct studies on smoking and its impact on public health. The group members were selected from these several ministerial departments: Health, External Relations, Agriculture, Trade, Finance, Communication, Education and Justice. In July 2005, “GROUPE”lobbied parliament to pass Law N°2005/005 [40] that authorized the President of Cameroon to ratify the FCTC, which was effectively ratified in February 2006. After the ratification, the Prime Minister, in order to mark Cameroon’s adherence to this framework agreement, convened a multisectoral meeting with all the Country’s ministerial departments and mandated each to develop policies for preventing and controlling smoking in their respective departments. Following this mandate, several ministries including Basic Education, Secondary Education, Higher Education, Finance and Social Affairs developed internal policies to establish smoke-free areas. Due to the will of actors of the Ministry of Secondary Education, the formulation of this policy was done easily, according to key informants. This rapid formulation of policy was alsoreported by the tobacco expert group responsible for formulating the policy requiring on health-label-warnings on cigarette packaging “GROUPE” then worked on the drafting of this conjoint decision between the health and trade sectors.Thereafter, “GROUPE” developed a national comprehensive law project on tobacco control based on the FCTC’s recommendations.This law revised in 2012 is still at the Presidency of the Republic in Cameroon and has not been presented to parliament for enactment.

### Actors

The major actors involved in formulating and implementing tobacco use prevention policies were ministerial departments of Health, Trade, Education, Communication and Finance. We mapped the major actors involved in formulating of some tobacco control policies according to their level of involvement and their position (Table 4). Interaction of different actors in the policy development and implementation process depended on whether the intervention had a multisectoral scope or not. For example, four interventions were multisectoral, whereas eight interventions involved only a single sector (principally sectors which adoptedsmoke-free indoor workplaces: Education, Finance, Social Affairs and one Sub division). Most of the main actors were the government sectors and it appeared from the analysis of the actors’ play in Cameroon.

**Table 4:** Actors involved on the formulation and implementation of some tobacco use prevention policies in Cameroon

Policy	Numbers of sectors involved	Types of sectors	Role in the policy process			Level/extent of involvement			Actor positions		
			Formulation	Implementation	Follow-up/Evaluation	High/leadership role	Middle	Low	Supportive	Mixed	Non-supportive
Law No. 2006/018 of December 29, 2006 governing advertising in Cameroon which prohibit advertising of tobacco products	7	Parliament	x			x			x		
		Presidency	x				x		x		
		Ministry of Communication	x		x	x			x		
		Town Hall		x			x		x		
		Tobacco industry representatives		x			x			x	
		Advertising companies		x			x		x		
		Ministry of Justice		x			x		x		
Law N° 2014/026 of 23 December 2014 on the finance law of the Republic of Cameroon for the 2015 financial year	8	Parliament	x			x			x		
		Presidency	x				x		x		
		Prime Ministry	x				x		x		
		Ministry of Finance/ General Direction of Taxation	x	x	x	x			x		
		Ministry of Finance/ General Direction of Customs		x			x		x		
		Ministry of Trade	x	x			x		x		
		Ministry of Health	x					x	x		
		WHO	x			x			x		
		GICAM [Inter Patronal Groupings of Cameroon]	x					x		x	
		Tobacco companies		x			x			x	
Order N°967/ Ministry of Public Health and Ministry of Trade of 25 June 2007 Health Warnings on Tobacco Products	5	Expert Group for Tobacco Control	x								
		Ministry of Health	x		x	x			x		
		Ministry of Trade	x	x	x	x			x		
		General Direction of Customs		x					x		
		Tobacco industries		x			x			x	
Circular No. 19/07/ MINESEC/SG/HR/SKSAPPS of 11 September 2007 on Creating smoke-free zones and anti-smoking clubs	2	Ministry of Secondary Education	x	x	x	x			x		
		LUTOMA [Association for the fight against drug addiction and mental illness]	x				x		x		

Informants cited political will, the existence of a formal exchange platform and personal leadership of the various actors in relation to awareness of the dangers of tobacco as factors facilitating cooperation of different sectors/actors at the level of formulation. The factors which contributed to the cooperation of actors between the different sectors in the implementation of these policies were respect of instructions from hierarchy and the adherence of stakeholders to the cause. Informants cited inadequate funds and lack of synergy between sectors as the main barriers.

### Policy implementation status

Tobacco use prevention and control policies were only partially implemented, with a high variability between policies and regions. Key informants said that policy implementation of increased taxes on cigarettes was fully effective immediately after the promulgation of the 2015 Finance Act. Since January 2015, the updated excise duties rates have been fully implemented.

About the smoke free zones at secondary schools, key informants at the central level of the Ministry of Secondary Education, estimated that about 80% of secondary schools had implemented the ministerial circular creating smoke-free zones with clearly visible message boards. However, we could not corroborate this level of implementation of our observations in the 5 regional capital cities we visited. We observed the signaling of schools as smoke-free zones with clearly visible message boards in about 50% of the schools in Yaoundé and Garoua, and only 10% in the other cities (Douala, Ebolowa, and Bamenda). Further, the message was not always exactly as on the circular. In some cases we saw only a no-smoking symbol: a cigarette overlaid by a red circle and cross**.** Regarding the second part of this policy, according to key informants, the creation of anti-tobacco clubs was not effective. Nevertheless, awareness activities were integrated into other existing clubs in schools such as the health club.They also thought there was a clear reduction in smoking prevalence in schools. Students no longer smoke on school premises, and teachers who did so in the past have stopped the practice. This assertion could not, however, be verified.

Implementation of the policy on banning of tobacco advertising, promotion and sponsorship was also observed not to be fully effective. In fact, we observed as mentioned by key informants, a 100% effectiveness in the official advertising sector; however we observed posters of cigarette adverts in unofficial environments such as on the walls of pubs, bars, informal drinking spots and shops in all the five cities.

Meanwhile, for the policy of health warnings on tobacco products, the extent of implementation was much more effective compared to the other measures. According to informants, this policy was almost 100% effective in the field. Any unmarked products in circulation would usually be contraband. The results from observations we conducted confirmed the information from informants; very few unmarked products werestill found in circulation and were probably contraband as they had indicated.

## Discussion

Explored through Walt and Gilson’s conceptual framework, Cameroon’s tobacco use prevention policies revealed how public action for tobacco use prevention and control takes shape in an environment of conflicting motivations. Indeed, the country is torn between the perceived need for public health to regulate tobacco consumption and the income its production, manufacture and sale generates. In 2006, public action for tobacco use prevention and controlwas amplified by the international mobilization and advocacy to enhance tobacco control. The WHO FCTC, ratified by Cameroon in 2006, coincided with the national reform of the health system, which set up a mechanism for the management of NCDs whose prevalence has steadily increased. But during the same period, the economic situation was problematic as Cameroon is a developing country that derives a part of its income from the tobacco production it promotes.

From 2006, prevention and control policies on tobacco use, which included two texts from the health and education sectors incorporating “best buy” interventions, was enriched with 10 texts reinforcing the coverage in the aforementioned sectors and in the Communication, Territorial Administration, Finance, and Social Affairs sectors. The contents of these 12 tobacco use prevention policies were based on the available evidence. Indeed they aligned at a certain level with the four tobacco prevention “best buy” interventions proving to be very efficient internationally: increasing taxes on tobacco, protecting people from tobacco smoke, banning tobacco advertising, and warning the public about the dangers of tobacco. However, in the midst of all these efforts made by the Cameroonian government, tobacco control has not yet resulted in uniform and integrated practices and their effectiveness and impact remains inconsistent. Considering taxes applied in tobacco products, they were only 26, 19 and 21% of the total retail price of the cigarettes respectively in 2010 [41], 2012 [1], and 2014 [42] and this is below the recommended proportion of 75% of retail price [42]. This is one of the lowest rates of levies on cigarettes in Africa and the world, far from the 80% of France and Madagascar. Despite the increase in the perceptible minimum which led to an average increase of 100 CFA francs per pack of 100 sticks of cigarettes, excise duties remain at 25% and this makes Cameroon one of the country with the lowest excise duties in the world, and taking into account smuggling, the black market, and especially the predominance of retail sales, this price is very low, making the cigarette very affordable [43].

In addition, Cameroon also has many policies incorporating the “best buy” intervention on protection against exposure to passive smoking, adopted in schools, in some ministries, and in the Sub-division of Bamenda I. But the impact of these policies is low due to the limited numbers of institutions fully implementing them. Smoking is still permitted in the general social space. Similarly, the policy banning tobacco advertising, promotion and sponsorship is only partially implemented and public posters promoting tobacco use remain in non-regulated areas. However, the policy on requiring health warnings on tobacco product packaging meanwhile is more universally applied consistently, with exceptions of illegally smuggled cigarettes. Nevertheless, its effectiveness remains limited in a country where the illiteracy rate is still a problem and where consumers are not often in contact with cartons of cigarettes because retail purchases of individual cigarettes are common in the streets [5].

Our examination reveals a discrepancy between urgency of the problem of tobacco use and the effectiveness of the corrective measures. In other words “best buy” interventions are integrated into Cameroon’s repertoire of tobacco prevention and control policies but are not completely applied, and therefore are of limited effectiveness, leading to a reduction of the potential positive effects and an apparent failure of the “best buy” interventions. Therefore, the reduction of the tobacco consumption level for both youths and adults was not observed as expected since these policies were adopted [44]. In 2003, the STEPS survey showed a prevalence of 6.3% of current tobacco smoking in people age 15 and more [41]. In the GATS 2013, a prevalence of 6% in the same age group was current tobacco smokers and 5.7% currently smoked manufactured cigarettes [5]. On the Global Youth Tobacco Survey done in 2008 and 2014 among school children aged 13 to 15 years, 5.7% currently smoke cigarettes [6, 9].

Yet it has been demonstrated that rapid implementation of the interventions described in the FCTC and above all emphasized by “best buy” interventions should have reduced smoking Disability Adjusted Life Year burden by 50% and also reduced the epidemic health effects, and economic cost [45–50]. Then, it appears that in Cameroon, the implementation mechanism of public action against tobacco is incomplete. Actual policy responses tend to be piecemeal and do not provide a comprehensive and integrated response to the problem of smoking. Furthermore, the absence of a comprehensive tobacco control bill, blocked at the Presidency of the Republic in Cameroon, is decried as being the likely linchpin for the insufficiencies in practice, policy and application of tobacco control in the country [51]. This situation is unfavorable for the tobacco prevention and control environment, especially since WHO recommended the integration of these policies across all government departments [10].

The major element that appears as facilitator and barrier to progress is related to the motivations of the actors in implementing public policy [52]. Decision makers’ dual interests (in both supporting tobacco farmers and supporting limited tobacco use) and inefficient mechanisms (absence of a national comprehensive and coordinated process on the adoption of tobacco use prevention and control policies) have limited effectiveness in both formulating and implementing “best buy” interventions. This study found that that at the beginning of the measures on tobacco, the WHO FCTC ratification mobilized a strong political will: Prime Minister convened a meeting and instructed each ministerial department to develop policies for smoking prevention and control in their respective departments. But the rest of the process has been characterized by slowness, passivity and even contradictions in government action. Although some ministries (health, education, finance, social affairs) followed the Prime Minister’s instruction, others did not follow up the directive, without any negative consequences.

Finally, the general political context is characterized by what Hardin [53] calls “general inertia”. He evokes the total dissolution of power and the absence of the power of coordination that induce the loss of efficiency, and the inability to produce results. This situation has been raised by the President of the Republic of Cameroon during several of his new year messages to the nation. In 2003, he said: “My fellow compatriots, as you can see; our main enemy is not the lack of resoources or even financial capacity. It is inertia. That is what we must fight if we have to move forward” [54]. In 2013, he made the same observation: “We have talented, resourceful, well-trained and enterprising men, women and youth, who are capable of meeting these challenges. We have abundant and diverse natural resources as well as modern and democratic institutions. Our country is peaceful and stable. What then do we lack?” [55]. Between “inertia” and conflicting interests linked to the profits generated by tobacco production [8], the government’s commitment is not complete.

In fact, the FCTC, recognizing that its recommendations will be detrimental to the farmers who depend on tobacco for their livelihood, encouraged governments to help tobacco farmers make the transition from tobacco to other crops [56]. Despite this, the government-which was supposed to be the main player in tobacco control-granted huge subsidies to tobacco farmers in the country [51]. Most of the main actors were the government sectors and it appeared there was a low visibility of civil society organizations and consequently the lack of collective mobilization on the issue of tobacco use. Cameroonian civil society remains very weakly mobilized for various reasons. It operates in a social context strongly marked by a recent history of the the monopoly of the State characterized by a unique thought process and the strong repression of all alternative thoughts. Also, civil society remains not very credible because of the opportunistic profile of its structures and the poor administrative and financial management methods imported from the public administration [57]. The balance of power between the government conflict of interest and the other actors engaged in tobacco control is heavily weighted in the government’s favor and the status quo.

### Current issues of tobacco use prevention and control in Cameroon

From the above, it appears that the policies currently in place are insufficient to solve the tobacco-use problem as shown by the stability of tobacco consumption at around 6% of both adolescents (13–15 years) and adults since at least 2003 [5–7, 9, 41]. As defined by Hall [58] and adapted by Muller [59], public policy change has three drivers: a change in the objectives of policies and, more generally, of the normative frameworks that guide public action; a change in the instruments that make it possible to implement public action; and a change in the institutional frameworks that structure the public action in the area concerned. On analysis, it appears that these three aspects have been initiated but not exhaustively established. Indeed, on the basis of factual data [8, 46, 60], it appears that a missing link to increase the effectiveness of the control of tobacco consumption is adequate and comprehensive legislation that established clear objectives, means of action and institutional frameworks to structure public action. In other words, tobacco use control is deadlocked pending law at the Presidency of the Republic, still in study according to the official version. But some NGO leaders think it is blocked by the pressure of businessmen whose interests would be threaten [59]. While this pending law would serve to institutionalize behaviors throughout the Cameroonian territory toward of tobacco control. To move forward, all parties will need to agree on effective regulation that has both (1) established appropriate actions and (2) stated negative consequences of not changing actions.

Nevertheless, a law is not the panacea, since “society is not changed by decree” [61]. It is evident that other neglected components would amplify effectiveness in controlling tobacco use. The existence of a law alone would not be enough to change practices with regard to tobacco but would also require a strong and active political will to translate regulation into practice. Chevallier [62] explains that in order to implement a regulation, the mobilization of a set of resources by the public authorities is required. A strong political will is therefore essential to lift the blockages and to overcome the resistance of the forces hostile to change including tobacco industry; and the involvement of administrative services is required to concretize the decisions taken. This strong political will must be led by the President of the Republic, who can firstly validate the anti-tobacco bill project, allowing it to be passed through the legislature. Secondly, the Government of Cameroon will have to put the necessary ressources for an effective implementation of the bill. The experience of South Africa, which is a successful example of tobacco control, shows that tobacco control requires the permanent contributions of different actors. South Africa has seen a significant decline in tobacco use, owing to the combined action of the government (with its comprehensive legislation and taking on the tobacco industry), the activism of civil society concerned by public health issues with community support [8], as well as research playing an essential role in supporting both policy development and advocacy. An inclusive collaborative framework is essential for effective tobacco control. Currently in Cameroon, the other element that emerges from the analysis of the actors’ play is the low profile of civil society organizations and the consequent lack of collective mobilization on the tobacco use issue. This situation is detrimental to the desired change and calls for innovative ways of involving communities in tobacco control.

## Limitations

The study was conducted over a limited time period. The results of the study should be considered as a “snapshot” capturing only a single point regarding stakeholders’views on tobacco use prevention policies and their implementation. These findings, therefore, suggest the need for correlated research for tobacco and other NCD precipitants. Despite these inherent limitations, this case study research method is essential in health policy research and then investigates a contemporary phenomenon within its real-life context. In such a setting, the boundaries between phenomenon and context are not clearly evident, and multiple sources of evidence must be used [13].

## Conclusions

This case study analyzing the context, content, and processes of formulating and implementing tobacco use prevention policies in Cameroon, based on document review, in-depth interviews and direct observations, provided a map of the situation. The findings revealed the existence of sectoral regulations integrating all the four WHO tobacco use prevention “best buy” interventions. All these policies are implemented with varying efficiency by policy and across regions. The policies on tobacco tax increases and health warnings had a high degree of implementation; those on protectingothers from tobacco smoke exposure on secondary schools and on banning tobacco advertising, promotion and sponsorship were onlypartially implemented.

These findings lead us to conclude that tobacco use control in Cameroon is at an impasse which calls for a change in public action on this issue. Consequently, important steps need still need to be taken to ensure the effectiveness and efficiency of all these tobacco use prevention “best buy” interventions. Improvement will require (1) the adoption of a comprehensive and integrated antitobacco law project, which contain a coherent, inclusive and incentivizing framework of action; (2) an increase of domestic budgetary allocations for the prevention and control of tobacco use and the exploration of viable financing options, including the increase of tobacco taxation: in Cameroon, the tobacco excise duty is 25% and it can be increased up to 75%; (3) an strengthening of the existing national multisectoral platform with a strengthening of civil society’s influence on processes and an extensive mobilization in the community. These actions are necessary to further decrease tobacco use, thereby reducing tobacco use related NCDs.

## Abbreviations

ANPPA: Analysis of non-communicable disease prevention policies in Africa
CEMAC: [Central African economic and monetary community]
CFA: [African financial community]
FCTC: Framework convention for tobacco control
GICAM: [Inter patronal groupings of cameroon]
LUTOMA: [Association for the fight against drug addiction and mental illness]
NCDs: Non-communicable diseases
USD: United States Dollar
WHO: World health organization
